# An identity crisis: the need for core competencies in undergraduate medical education

**DOI:** 10.3402/meo.v18i0.21028

**Published:** 2013-04-29

**Authors:** Jeffrey B. Russ, Anna Sophia McKenney, Ankit B. Patel

**Affiliations:** Weill Cornell/Rockefeller/Sloan-Kettering Tri-Institutional MD-PhD Program, New York, NY, USA

**Keywords:** undergraduate medical education, core competency, Flexner II

## Abstract

A medical student perspective on the role of core competencies in undergraduate medical education in light of medical education reform associated with recent Flexner II.

There is no question – it is exciting to be a medical student in the era of Flexner II. No longer confined to the simple ‘two-plus-two’ system, our educational schemata are evolving at a blinding pace. Basic science and clinical curricula are merging into one. Areas of concentration and tracks of individualization are giving us the opportunity to become medical professionals with a special edge, and condensing certain curricular components will help some students more efficiently expedite a long, grueling training path in order to pursue these individually enriching training experiences. These changes are breaking open a previously confining paradigm of education and are sure to be a springboard for students to become a diverse set of medical experts.

However, this movement also inspires some trepidation. In particular, these changes call into question the real nature of medical education and the role of doctors as a whole. As we trim (or densely compress) our medical training to allow for additional experiences, we are also forced to examine the core that remains. What does it *really* mean to be a doctor? When I graduate, will I have the same knowledge and skills as my new colleagues from other schools? And what should be my role, as an MD, on a team of diverse medical professionals with apparently similar training? For our education to meet its full potential, it is not enough to know what makes us individuals – we need to understand what important aspects of our education are at the core, defining our identity as doctors.

To this end, many medical organizations have turned to standardized competencies for medical training, a strategy that has had a rich and controversial history over the last few decades (1). Most notably, the Accreditation Council for Graduate Medical Education (ACGME) defined six major competencies they deem necessary for residency training: patient care, medical knowledge, professionalism, systems-based practice, practice-based learning and improvement, and interpersonal and communication skills (2). These core competencies have transformed the framework by which residency programs evaluate their residents, and importantly, core competencies have unified residency programs nationally by the values instilled in their trainees.

To some degree, this initiative has shaped the agenda for undergraduate medical education as well; for instance, the Liaison Committee on Medical Education (LCME) now requires medical schools to outline their curricula in terms of objectives that will allow students to demonstrate achievement of certain competencies (3). Currently, however, medical schools are left to their own devices to determine what core values they strive to imbue in their students, and as many medical schools are now refreshing their educational programs in the neo-Flexnerian spirit, new curricular reforms and innovation are creating significant variability between medical schools. An attempt to unify medical schools’ training goals was undertaken in 1998, when the AAMC drafted a list of 30 curricular objectives, each categorized under the four umbrella values of being altruistic, knowledgeable, skillful, or dutiful (4). However, this now 15-year-old list served mainly as a curriculum content design tool rather than as a nationally agreed upon delineation of what might be desired in a modern graduating medical student. We believe that graduate medical education benefited from reflecting on a single set of universal competencies for residents, and undergraduate medical education could similarly benefit from undertaking such an exercise, in order to again clarify the core values of the medical student in this newly reformed era of medical education.

What would such a list of core competencies for medical students look like? While it might overlap with or be modeled after the competencies delineated by the ACGME, the undergraduate list would necessarily include competencies that are distinct for the medical student. For example, theoretical aspects of scientific inquiry and mechanistic exploration might be major undergraduate medical competencies, while more practical elements, such as complex procedural competency, could be deemphasized in favor of later instruction. Additionally, we must be mindful that overuse of the ACGME list of competencies to drive individual assessment has been a source of discontent (1, 5, 6). We envision, instead, that a consensus list of core competences could create a more standardized ‘mission statement’ to help medical schools anchor their curricular reform processes around shared values and to help students in the development of their professional identity.

As medical students anticipating the interdisciplinary future of medicine, we welcome the reforms of Flexner II aimed at diversifying the undergraduate medical education experience. We acknowledge the fundamental role of undergraduate medical education in preparing us for our future as physicians, and more immediately as interns and residents, and are dedicated to protecting the core tenets, or core competencies of our education. We feel the core competencies of undergraduate medical education should be defined universally and be used to guide new curriculum structures. Once created, these core competencies would help to preserve a nationally standardized foundation upon which medical education reform can ultimately mold the future physician workforce.

## References

[1] Lurie SJ (2012). History and practice of competency-based assessment. Med Educ.

[2] Accreditation Council for Graduate Medical Education Common program requirements. Approved 2010. http://www.acgme.org/acgmeweb/Portals/0/dh_dutyhoursCommonPR07012007.pdf.

[3] Liaison Committee on Medical Education Functions and structure of a medical school: standards for accreditation of medical education programs leading to the M.D. Degree. http://www.lcme.org/functions2011may.pdf.

[4] Anderson MB, Cohen JJ, Hallock JE, Kassembaum DG, Turnbull J, Whitcomb ME (1998). Association of American medical colleges Report I learning objectives for medical student education: guidelines for medical schools.

[5] Lurie SJ, Mooney CJ, Lyness JM (2009). Measurement of the general competencies of the accreditation council for graduate medical education: a systematic review. Acad Med.

[6] Wear D (2009). A perfect storm: the convergence of bullet points, competencies, and screen reading in medical education. Acad Med.

